# Iron and cancer: overview of the evidence from population-based studies

**DOI:** 10.3389/fonc.2024.1393195

**Published:** 2024-08-23

**Authors:** Rola S. Zeidan, Hyung-Suk Yoon, Jae Jeong Yang, Amin Sobh, Dejana Braithwaite, Robert Mankowski, Christian Leeuwenburgh, Stephen Anton

**Affiliations:** ^1^ Department of Physiology and Aging, College of Medicine, University of Florida, Gainesville, FL, United States; ^2^ Department of Health Outcomes and Biomedical Informatics, College of Medicine, University of Florida, Gainesville, FL, United States; ^3^ Cancer Control and Population Science Division, University of Florida Health Cancer Center, Gainesville, FL, United States; ^4^ Division of Hematology and Oncology, University of Florida Health Cancer Center, Gainesville, FL, United States; ^5^ Department of Surgery, University of Florida College of Medicine, Gainesville, FL, United States; ^6^ Department of Clinical and Health Psychology, University of Florida, Gainesville, FL, United States

**Keywords:** iron, diet, physiology, cancer, epidemiology

## Abstract

Iron is an essential nutrient required for various physiological processes in the body. However, iron imbalance can potentially contribute to initiating and promoting cancer development. Epidemiological studies have investigated the relationship between dietary iron intake and the risk of different types of cancer, yet, not all studies have consistently shown a significant association between dietary iron and cancer risk. Also, studies have shown different effects of dietary heme and non-heme iron intake on cancer risk. While some epidemiological studies suggest a possible link between high dietary iron (mainly heme-iron) intake and increased cancer risk, the evidence remains inconsistent. Moreover, multiple iron biomarkers, which can mirror physiological iron status, have demonstrated varied correlations with the risk of cancer, contingent upon the specific biomarker analyzed and the type of cancer being investigated. Here, we have investigated the current evidence on the potential relationship between dietary iron intake on one hand, and iron biomarkers on the other hand, with the risk of developing different types of cancer, including breast, prostate, lung, pancreatic, colon, colorectal, and liver cancers. Further research is warranted to better understand the complex relationship between dietary iron, physiological iron and cancer development. Future research should account for factors that affect and interact with dietary iron and physiological iron levels, such as genetic susceptibility, overall diet quality, and lifestyle habits.

## 1 Introduction

Iron is an indispensable mineral that plays crucial roles in multiple physiological processes including oxygen transport and DNA synthesis (1). Given the importance of iron for proper functioning of cells and organs, iron levels need to be maintained within recommended ranges. Iron imbalance, whether it is an overload or a deficiency, can severely impact normal biological processes (2) and induce several pathologies (3, 4). One of the detrimental effects of cellular iron overload is cancer, where iron can induce free radicals that can initiate and promote carcinogenesis (5). Similarly, iron deficiency has been shown to promote cancer progression. Anemia for instance has been associated with several types of cancers including cervical, colorectal and gastrointestinal cancers (6–8). Since the human body lacks a regulated excretory pathway for excess iron, dietary iron absorption is a critical factor in maintaining physiological iron levels (1). Iron balance is primarily regulated through the regulation of intestinal iron absorption from dietary sources (9). Importantly, physiological iron levels in the body are influenced by dietary iron consumption, as the body absorbs iron from food to maintain optimal levels (1). Even though iron can be recycled, the body requires a consistent supply of iron to meet its metabolic needs and maintain adequate iron stores and replenish daily iron losses (1). This supply of iron is provided by dietary iron intake. Dietary iron exists as heme iron from animal products, which is highly bioavailable and essential for older adults due to absorption issues, and as non-heme iron which is mostly from plant-based sources, with heme iron being the bigger contributor (4). Although heme iron constitutes a smaller portion of dietary iron, it is absorbed more efficiently and has a higher bioavailability than the non-heme iron (4). Non-heme iron absorption can be affected by factors such as duodenal pH and its intestinal absorption can be enhanced by co-consumption of vitamin-C rich foods (4). Thus dietary iron intake directly influences physiological iron levels (1). Therefore, a balanced dietary iron intake would help maintain normal physiological iron levels (1, 4). For more details, the readers are prompted to refer to our review titled “Iron homeostasis in older adults: balancing nutritional requirements and health risks” (4).

**Figure 1:**
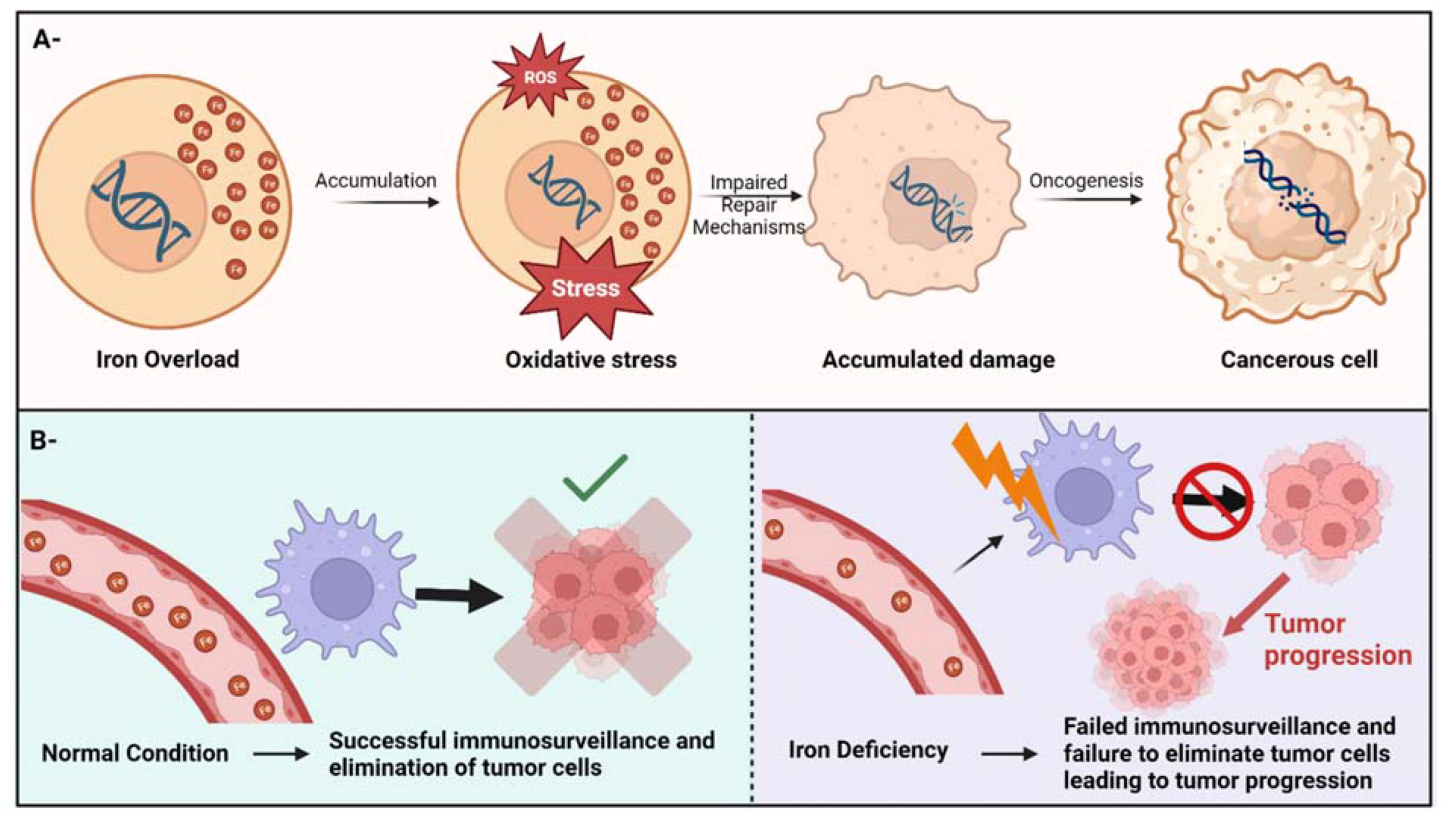
Iron imbalance can cause cancer through different mechanisms. **(A)**- the potential mechanism of iron overload-induced cancer through oxidative stress. **(B)**- Iron deficiency can lead to cancer by compromising the immune system’s surveillance, resulting in the inability to detect and eradicate cancer cells, consequently facilitating cancer progression.

Iron imbalance can occur in two ways: overload or deficiency. While iron overload is characterized by excessive accumulation of iron in the body, iron deficiency occurs when there is an insufficient amount of physiologically available, circulating iron (10). Iron overload has been shown to promote tumor growth by providing a favorable environment for cancer by several mechanisms such as inducing oxidative DNA damage (11), which can lead to mutagenesis and carcinogenesis. Additionally, excess iron can generate reactive oxygen species that can result in damaging cellular components, mainly lipids and DNA – **Figure 1A**. Lipid peroxidation leads to mitochondrial damage and dysregulation of mitophagy, both of which are implicated in carcinogenesis and tumor progression (12, 13). Mechanistically, ferrous iron (Fe^2+^) can react with hydrogen peroxide to produce a hydroxyl radical through the fenton reaction (14). The produced hydroxyl radical can further react with superoxides (a byproduct of cellular respiration) to produce larger amounts of hydroxyl radicals through a cycle known as the Haber–Weiss reaction (14).

Iron deficiency on the other hand, can jeopardize the immune system’s surveillance ability to detect and eliminate cancer cells, therefore facilitating tumor progression (15) – **Figure 1B**. Also, anemia due to iron deficiency (a condition that usually develops from long-term or severe iron deficiency) has been linked to DNA damage through several potential and unverified mechanisms (16). Additionally, insufficient iron levels can impair DNA repair mechanisms since iron is an essential cofactor for several enzymes that play an essential role in DNA replication, DNA repair, and cell cycle progression (17). Notably, women and girls in the reproductive years are at a higher risk of developing iron deficiency anemia due to the menstrual cycle, potentially increasing their risk for certain cancers (18).

In general, dietary iron intake (both heme iron and non heme iron, although the evidence for heme iron association with cancer appears to be more prominent) has been associated with an increased risk of several types of cancer including, breast, prostate, lung, pancreatic, colon and colorectal, as well as liver cancers (14). Notably, familial genetic factors, such as variations in iron metabolism genes (e.g., HFE mutations), can influence iron absorption and storage, leading to higher iron levels that generate reactive oxygen species (ROS) and increase the risk of cancer (19, 20). Despite the growing body of evidence linking both iron overload and deficiency to cancer, these findings primarily stem from epidemiological studies. Systematic confirmation of these observation remains challenging due to the intricate redundancy in the mechanisms that maintain iron homeostasis.

In most population-based research to date, measuring dietary iron intake (i.e., total, heme, and non-hem iron) or biochemical iron markers has typically been used as a proxy for determining an individual’s iron status. However, there remains substantial debate about whether dietary iron contributes to physiological iron status, as well as risk of cancer development and progression. Here we briefly describe findings of epidemiological studies examining the association between dietary and physiological iron with different types of cancer.

## 2 Methodological considerations

As this is a narrative review, articles were selected based on their relevance to the topic rather than strict inclusion and exclusion criteria. Although our review is not systemic in nature, a search strategy, described below, has been utilized to find relevant literature. Pubmed was searched using suitable keywords for each section, and the selected keywords were based on the following considerations: 1) the medical and scientific terms pertaining to the focus of each section, 2) the population of cancer patients (focusing on adults not pediatric patients), and 3) the research methodology used, focusing mainly on meta-analyses of the literature, as well as case-control, cohort and epidemiological studies. The search was unrestricted by publication date, with higher emphasis on more recent publications. Only English-language articles were considered. The search was driven by content; however, inclusion criteria encompassed studies offering valuable insights, theoretical frameworks, or empirical evidence pertinent to the correlation of dietary and physiological iron with cancer risk. Studies lacking relevance or methodological rigor were excluded based on the authors’ judgment. Additional references were sourced from the literature cited.

## 3 Dietary iron and cancer

After 16 years of follow-up for 536,969 middle-aged and older Americans, the NIH-AARP Diet and Health Study reported that heme iron intake was significantly associated with a 10%-15% increase in cancer (all types) incidence. Interestingly, epidemiological evidence indicates that top non-gender specific cancers correlated with dietary iron intake are GI cancers (specifically colon and colorectal) and lung cancers (21). Several studies concluded that correlation between dietary iron intake and cancer risk depends on the type of dietary iron. Heme iron specifically can act as a nitrosating agent, forming compounds that are known to be carcinogenic (21). For instance, while total iron consumption may lower the risk of esophagus cancer, heme iron intake may increase the risk of developing it (22). Other studies have investigated the association between dietary iron intake and different types of cancer as shown below.

### 3.1 Colon and colorectal cancer

Dietary iron has been shown to affect the expression of tumor specific genes (specifically adenomas polyposis coli or APC) and can induce *KRAS* (Kirsten rat sarcoma viral oncogene homolog) mutations, promoting the risk of colon and colorectal cancers (23). A meta-analysis by Fonseca-Nunes et al. incorporating 59 epidemiologic studies published between 1995 and 2012 reported that an increase of 1 mg/day in heme iron intake was associated with an 8% increased risk of colorectal cancer and 12% increase in colon cancer (21). In contrast, a meta-analysis of 9 case-control or cohort studies reported a 17%-27% decreased risk of colorectal cancer with dietary iron intake (and supplemental iron intake), but a 23% increased risk associated with dietary heme iron (24). The European Prospective Investigation into Cancer and Nutrition (EPIC) study also showed a 20% risk reduction in colorectal cancer associated with dietary non-heme iron only among men, indicating a potential sex-specific association between iron intake and cancer risk (25). On the other hand, a recent study revealed no association between total (heme and nonheme), heme or non-heme iron intake with the risk of developing colorectal cancer in women, and a similar, with non-significant association of heme with colorectal cancer in men (25). Additionally, the same study showed an inverse association of non-heme iron intake and colorectal cancer only in men (25).

### 3.2 Other GI tract cancers

A pooled analysis of the stop consortium revealed that total dietary iron intake was inversely associated with gastric cancer, and that adjustment by meat and fruit/vegetable intake had no effect on the results (26). A dose-response meta-analysis of five studies revealed that an increase of 5 mg/day in total iron intake was associated with a 15% reduction in esophageal cancer risk, but an increase of 1 mg/day in heme iron was associated with a 21% risk increase (27). A recent pooled analysis of 11 case-control studies found a 36%-37% risk reduction associated with dietary iron intake and gastric cancer risk (26). In addition, the NIH-AARP study of 322,846 older Americans who were followed-up for an average of 9.2 years showed a non-significant positive association between iron intake and pancreatic cancer (28), but when restricted to heme iron from red meat, a 32% increased risk of pancreatic cancer was observed among men.

### 3.3 Lung cancer

Lung cancer patients have elevated levels of interleukin-6 (IL-6), which can upregulate hepcidin (29) (a systemic iron availability regulator – for more information on iron homeostasis regulation, refer to our review titled “ Iron and Organismal Aging” (1)). Hepcidin can decrease systemic iron bioavailability, inducing cancer-related anemias, and also increasing the cellular iron load, increasing cellular redox stress, further contributing to cancer risk (29). Dietary iron intake has been correlated with an increase in the risk and incidence of lung cancer. The evidence on the association between heme iron consumption and the risk of lung cancer has been inconsistent, however. As such, Fonseca-Nunes and colleagues’ meta-analysis revealed that an increase of 1 mg/day in heme iron consumption was associated with a 12% elevated likelihood of developing lung cancer (21). Similarly, the EPIC study found that after controlling for smoking history and other risk factors, heme iron intake was associated with a 16% increased risk of lung cancer (while non-heme iron was associated with a 10% decreased risk of lung cancer) (30). Contrarily, the Rotterdam Study (a prospective cohort study of 5,435 adults aged ≥55 who were followed-up for 22 years) showed a 42% decrease in lung cancer risk in relation to heme iron intake (31). Meanwhile, a meta-analysis of five lung cancer studies yielded a null association between heme iron intake and lung cancer risk (32). Similarly, a recent study revealed no association between dietary iron intake (total heme and non-heme) and lung cancer in the population analysed (33). However, the same study indicated gender-specific positive association between heme iron intake and lung cancer risk only in women (25).

### 3.4 Gender specific cancer

#### 3.4.1 Female-specific cancers

Interestingly, elevated iron levels can affect estrogen metabolism, contributing to breast tumor initiation, and alteration of iron metabolism in macrophages foster breast tumor progression (34). Several studies indicated a deleterious effect of increased dietary heme iron on breast cancer incidence risk (35). The meta-analysis by Fonseca-Nunes et al. mentioned above, reported that an increase of 1 mg/day in heme iron intake was associated with a 3% increased risk of breast cancer (21). Another meta-analysis of 16 breast cancer studies showed a 12% increased risk due to heme iron intake but no associations with total dietary or supplemental iron intake (36). As for endometrial cancer, a study has revealed a slight favorable correlation between dietary heme iron, overall iron, and liver consumption, and the risk of developing endometrial cancer. Conversely, the same study revealed no notable statistically significant connections found between the consumption of other types of red and processed meats and the risk of endometrial cancer (37).

#### 3.4.2 Male-specific cancer

In terms of prostate cancer risk, the EPIC study did not find a significant association for heme iron intake (38), while the NIH-AARP Diet and Health Study, analyzing 175,343 US men aged 50-71 years, showed a 9%-28% increased risk in relation to heme iron intake depending on stage (39).

### 3.5 Synopsis

**Figure 2:**
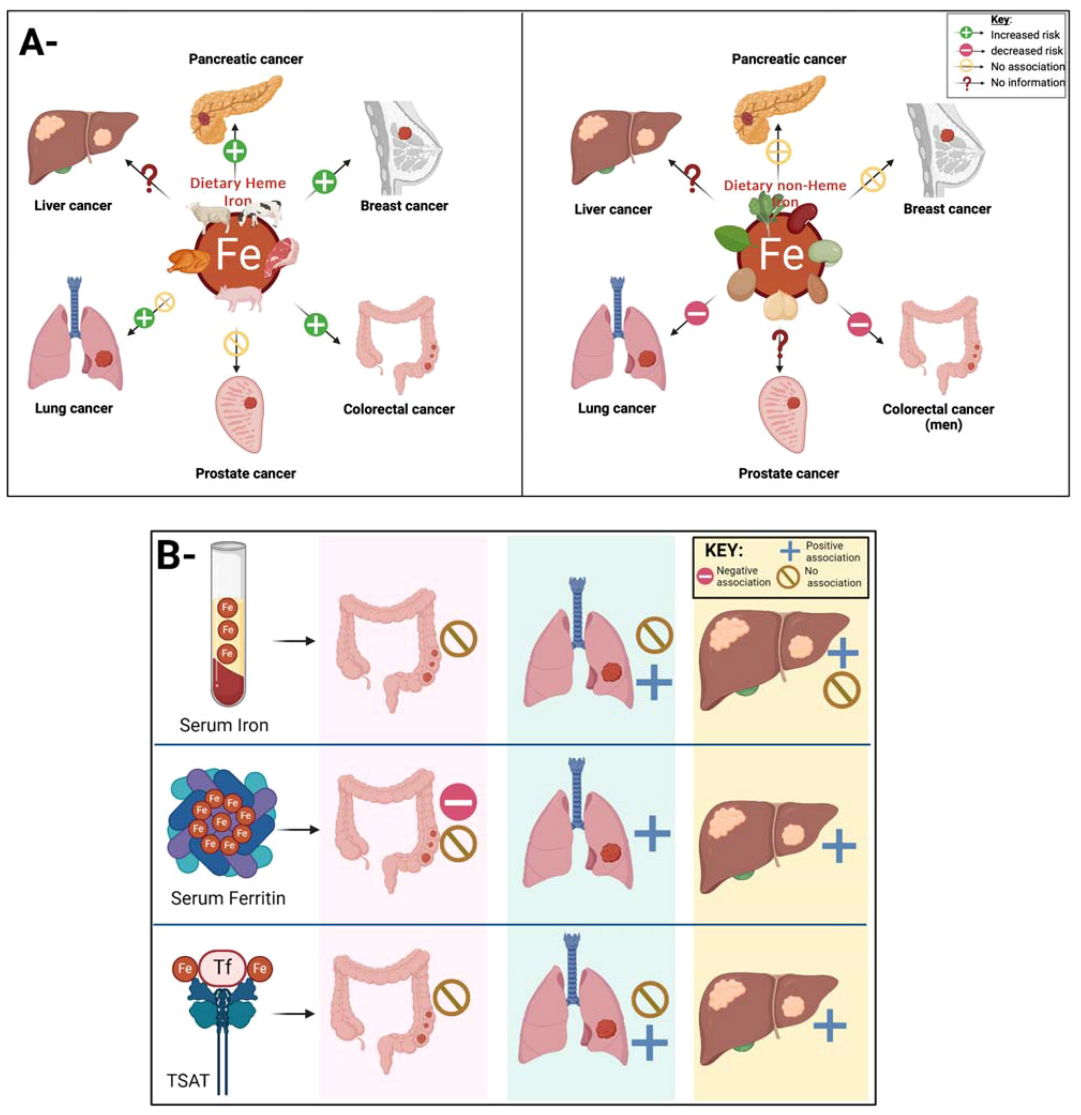
**(A)-** The intriguing link between dietary iron and cancer remains a topic ripe for investigation. Epidemiological studies can help investigate the current link between dietary iron intake and cancer. Current findings indicate a positive association between heme iron intake (panel A left) and colorectal, breast and pancreatic cancer, no association between heme iron intake and prostate cancer, and conflicting evidence with lung cancer. As for non-heme iron intake (panel A right), studies have revealed a reduction in lung and colorectal cancer, no association with the risk of breast and pancreatic cancer but no evidence on the link between non-heme iron intake and liver and lung cancer. **(B)**- Figure showing the link between different iron biomarkers and the risk of different types of cancer. Current evidence reveal no association between serum iron and TSAT with colorectal cancer, yet as for serum ferritin, the evidence of its correlation with colorectal cancer remains conflicting. This is similar to the association lung cancer with serum iron levels and TSAT where different studies showed conflicting results. The evidence is more consistent for liver cancer, where serum ferritin and TSAT were correlated with an increased risk, where as study sesults on the correlation between serum iron levels and liver cancers were inconsistent.

Taken together, a positive association between heme iron intake and several types of cancers was observed. On the other hand, non-heme iron intake was associated with a reduced risk of lung cancer (in both sexes) and colorectal cancer in men. **Figure 2A**. Thus, it is hard to define the exact impact of dietary iron intake on carcinogenesis and tumor progression yet from an epidemiologic angle. For instance, while Fonseca-Nunes et al.’s meta-analysis revealed an association of increased dietary iron intake and the cancers mentioned above, including colon, colorectal, breast and lung cancers, they found no significant association with other cancers due to the lack of relevant studies and heterogeneity across studies reported. The inconsistencies observed might be partially explained by variations in dietary assessment, measurement errors, and heterogeneity across baseline characteristics of the study participants. Additionally, gender difference in the correlation between dietary iron intake and the risk of certain cancers exists but is not always prominent, necessitating further focused investigation to better understand the gender differences of dietary iron-induced risk of cancer. A large-scale, prospective investigation incorporating a precise method for diet assessment and specific types of cancers is required to provide evidence of the potential application of dietary iron as a novel, modifiable factor for cancer prevention.

## 4 Physiological iron status and cancer

In addition to dietary iron intake, iron status can be assessed by measuring blood levels of total iron, transferrin, transferrin saturation (TSAT), total iron-binding capacity, and ferritin. Serum iron is known as a less specific marker for body iron stores, but ferritin and TSAT have been recognized as excellent indicators of body/tissue iron stores (21, 40). Some population-based studies have evaluated the association of these iron biomarkers with cancer, but no conclusions have been reached yet. Here we present the results of meta-analysis studies that assessed the association between specific blood iron markers (total iron, transferrin, transferrin saturation (TSAT), total iron-binding capacity, and ferritin) and different cancer types.

### 4.1 Colon and colorectal cancer

Both iron overload and iron deficiency have been implicated in colorectal cancer (41). A meta-analysis of four observational studies found the null associations for serum ferritin, serum iron, and TSAT with colorectal cancer (24). Meanwhile, in a nested case-control study (130 colorectal cases and 260 matched controls) within the Cancer Prevention Study, serum ferritin was associated with a 60% decreased risk of colorectal cancer (42). Colon cancer also showed an 80%-90% risk reduction in relation to serum ferritin, serum iron, and transferrin saturation, but a 4.7-fold increased risk with unsaturated iron binding capacity (42). Similarly, a study in Korea showed an inverse association between serum ferritin levels and the risk of developing colorectal cancer (43). Also, a large case cohort study revealed that lower levels of plasma ferritin are associated with an increased risk of gastrointestinal cancer (44). A nested case-control study within EPIC-Heidelberg Study, including colorectal cancer, indicated that serum iron, transferrin, or TSAT were not associated with risk of colorectal cancers, as well as cancer mortality. Similarly, a recent study revealed no association between liver iron content and colorectal cancer risk (45).

### 4.2 Lung cancer

Some lung cancer patients revealed high levels of serum ferritin (29). A meta-analysis by Ramirez-Carmona et al., of observational studies comparing serum ferritin levels between healthy adults and cancer patients reported higher ferritin levels in lung cancer patients (Standardized mean difference=1.72). Similarly, a meta-analysis of case-control studies showed significantly higher levels of serum ferritin and TSAT among lung cancer cases (SMD=0.235 and SMD=0.07, respectively), but significantly lower levels of serum transferrin concentrations (SMD=-0.591) (32). A nested case-control study within EPIC-Heidelberg Study, including lung cancer and other cancer types (see below) reported that serum iron, transferrin, or TSAT were not associated with risk of lung cancer development. On the other hand, a more recent study on the correlation of serum iron with the risk of lung cancer, revealed a causal correlation between serum iron, ferritin, transferrin, transferrin saturation and lower risk of lung squamous cell carcinoma, potentially suggesting a protective effect of iron (46).

### 4.3 Liver cancer

A meta-analysis of nine nested case-control or cohort studies showed that liver cancer risk was raised by 1.49 - to 2.47-fold increases in iron biomarkers such as serum ferritin and serum iron with substantial heterogeneity across studies (47). Similarly, a large case cohort study showed that iron overload as well as higher levels of serum ferritin are associated with an increased liver cancer riks (44). A large cohort study of 309,443 Taiwan adults showed that high serum iron levels were linked to a 2.98-fold increase in the risk of developing liver cancer (48). However, a nested case-control study showed no association between serum iron levels and primary liver cancer incidence (49). As for TSAT, it has been correlated with an increase in the risk liver cancer in both men and women (50), as well as in increasing the risk of hepatocellular carcinoma, particularly in people with a non-alcoholic fatty liver disease (51).

### 4.4 Other types of cancer

The meta-analysis mentioned above by Ramirez-Carmona et al. reported higher ferritin levels among all cancer patients (standardized mean difference [SMD]= 3.07, 95% CI=1.96-4.17), especially for head and neck cancer (SMD=3.88), pancreatic cancer (SMD=6.79), and renal cell carcinoma (SMD=1.77) (52). This study also revealed much higher ferritin levels among cancer patients with advanced stages than among cancer-free adults (SMD=4.89 for stage III and SMD=8.40 for stage IV), indicating the potential of ferritin as a cancer biomarker. Similarly, another study revealed that high serum ferritin levels are associated with an increased risk of pancreatic cancer (53).

A meta-analysis of 11 prospective studies investigating the associations between iron markers and breast cancer risk found a 22% increased risk associated with serum/plasma iron, but not for ferritin, transferrin saturation, or total iron-binding capacity (36). A nested case-control study within EPIC-Heidelberg Study, including breast cancer (n=627), prostate cancer (n=554), and cancer deaths (n=759), reported that pre-diagnostic serum concentrations of ferritin were associated with a 30%-33% decreased risk of developing or dying from breast cancer, but serum iron, transferrin, or TSAT were not associated with risk of prostate cancer as well as cancer mortality (54). On the other hand, a recent analysis from the NHANES survey data revealed an association between serum soluble transferrin receptor levels with male- and female-specific cancers of prostate, testis, breast, cervix, ovary and uterus (55).

In the Taiwan cohort study mentioned above, low and high levels of serum iron (<60 μg/dL or ≥140 μg/dL) were associated with a 18%-37% increased risk of developing any kinds of cancer and a 30% to 62% higher risk of dying from any cancers, highlighting a J-shaped association between serum iron and cancer (48). Furthermore, a study on nasopharyngeal cancer patients indicated an abundance of functional iron deficiency in the studied group, with serum iron, total iron binding capacity and transferrin being significantly lower than in their healthy counterparts (56). In addition, the Second National Health and Nutrition Examination Survey including 3,000 men and 3,244 women found a 82%-86% increase in cancer mortality associated with serum iron and TSAT (57). Also, interestingly, a recent study revealed that hair iron levels (although not particularly physiological), are significantly associated with the risk of esophageal squamous cell carcinoma (58).

### 4.5 Synopsis

While some studies suggest potential associations between certain physiological iron biomarkers and the risk of cancer development, results vary among cancer types (**Figure 2B**). For colon and colorectal cancer, mixed findings were observed with serum ferritin, iron, and transferrin saturation. In lung cancer, higher ferritin levels were consistently found among patients, but the correlation between other iron biomarkers and lung cancer was not consistent. Liver cancer risk was raised with elevated serum ferritin and iron levels. Additionally, elevated ferritin levels were noted across various cancer types, particularly in advanced stages. However, breast cancer risk was associated with increased serum/plasma iron levels but not ferritin or transferrin saturation. These findings underscore the complex relationship between different iron biomarkers and the risk of different cancer developments, necessitating further research to elucidate underlying mechanisms and clinical implications.

## 5 Discussion

Overall, dietary heme iron intake levels have been associated with several types of cancer, some more prominently than others. Specifically, dietary intake of heme iron has been consistently associated with colorectal, pancreatic and breast cancers but less consistently with prostate and lung cancers. On the other hand, dietary non-heme iron intake levels have been associated with a decreased risk of lung and colorectal cancers. Interestingly, the association between dietary iron intake and the risk of cancer appears to be gender-specific in some instances, warranting further research. Physiological blood iron markers, however, were less consistently associated with different cancer types. For instance, higher ferritin levels but lower transferrin levels were associated with an increased risk of lung cancer.

**Figure 3:**
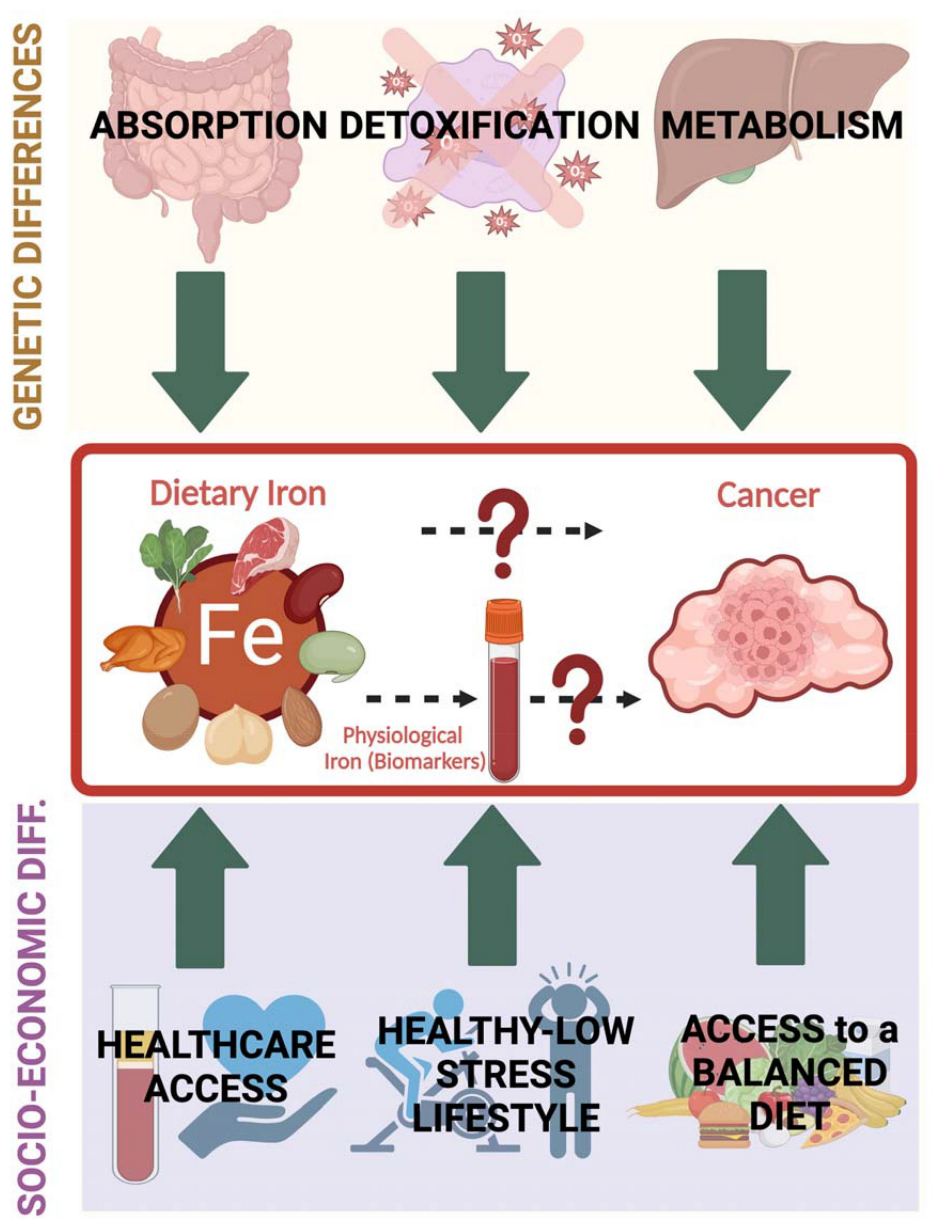
Individual variations that can influence the interplay between dietary iron (and physiological iron reflected by iron biomarkers) and cancer encompass multiple factors that can be mainly grouped into genetic (affecting absorption, metabolism and detoxification of iron-induced radicals) and socioeconomic (including access to proper health care and ability to maintain a balanced diet and a healthy lifestyle). Healthcare access allows periodic iron level assessment and reduces the risk of having unattended confounded health issues.

The inconsistencies observed in previous studies might be due to study design limitations including insufficient sample size, residual confounding, or heterogeneity in measurement methods. To fill research gaps and unmet needs, further studies are warranted to conduct a comprehensive evaluation of specific iron biomarkers after controlling for individual variabilities including clinical conditions as well as genetic and socioeconomic factors that can influence genetic susceptibility, overall diet quality, and lifestyle habits (**Figure 3**).

Although findings from epidemiological studies suggest there is a relationship between physiological iron and cancer, the detailed mechanistic relationship between iron and cancer is yet to be elucidated. Despite the biological plausibility between iron and cancer, epidemiological studies have failed to provide consistent evidence of the relationship, perhaps due to methodological limitations. Specifically, epidemiological studies on dietary iron, physiological iron biomarkers, and cancer risk face several limitations and challenges. Heterogeneous study designs, measurement errors (especially in dietary intake and iron biomarkers), confounding factors such as genetics, lifestyle, and presence of comorbidities, in addition to variability in biomarkers levels that can be transiently affected by multiple factors, can complicate the interpretation of results. Additionally, comparing diverse studies is challenging due to differences in populations, dietary assessment tools, definitions, thresholds, and publication bias. These issues necessitate standardized methodologies, thorough adjustment for confounders, and cautious interpretation to draw reliable conclusions. Addressing these factors is crucial for an unbiased analysis of the current evidence for understanding the association between dietary iron, iron biomarkers, and cancer risk.

Furthermore, while studies have provided more insights on the effect of iron overload on cancer initiation and progression (**Figure 1**), limited information is available on how iron deficiency may cause cancer, even though studies have suggested that iron deficiency anemias can potentially increase the risk of cancer (59, 60). Besides, the newly characterized iron-dependent cell death, known as ferroptosis, appears to play a dual and pivotal role in cancer where it can act as a tumor suppressor in several solid tumors, and even can inhibit the migration and invasion of cancer cells, and metastasis reduction, yet ferroptosis can also act as carcinogen in certain conditions (61). Therefore, it is proving to be a promising potential therapeutic target to regulate tumor progression and reverse resistance to cancer therapies (55, 62), providing more evidence on a plausible and important interplay between physiological iron and cancer. Collectively, the overall connection between dietary iron intake (differentiating between heme and non-heme iron), physiological iron levels (reflected by different iron biomarkers) and cancer is worth special scrutiny due to its capacity to shed light on the impact of food intake on illnesses including cancer, both of which can contribute to cancer disparities (5, 63, 64). This is especially relevant as particular ethnic groups and individuals from disadvantaged socioeconomic backgrounds are recognized for their susceptibility to both iron imbalances and increased cancer risks. Additionally, a better understanding of the relationship of iron and cancer can offer clinical potential for treating cancer. Several studies have already suggested targeting iron metabolism for treating different types of cancer, including osteosarcoma (65). Ferroptosis, as previously mentioned, has been heavily suggested as a promising cancer therapeutic target (66). A group of researchers has also suggested that modulating iron physiology, especially in the tumor microenvironment by iron deprivation and iron overload toxicity, with exercise, can be used to treat cancer patients (67). Furthermore, research evidence suggest the successful use of iron biomarkers as prognostics for certain types of cancer (68). All this warrants further research to better employ iron physiology in cancer therapies.
